# l-Alanine hydrochloride monohydrate

**DOI:** 10.1107/S1600536808008751

**Published:** 2008-04-04

**Authors:** Kazuhiko Yamada, Akira Sato, Tadashi Shimizu, Toshio Yamazaki, Shigeyuki Yokoyama

**Affiliations:** a National Institute for Materials Science, 3-13, Sakura, Tsukuba 305-0003, Japan; b Protein Research Group, Genomic Sciences Center, Yokohama Institute, RIKEN, 1-7-22 Suehiro, Tsurumi, Yokohama 230-0045, Japan

## Abstract

Colorless crystals of l-alanine hydrochloride monohydrate, C_3_H_8_NO_2_^+^·Cl^−^·H_2_O, were obtained from a powder sample that had been left standing in a refrigerator for a few years. The structure displays several inter­molecular hydrogen bonds: the hydroxyl O atom is involved in a single hydrogen bond to the chloride anion, while the ammonium group forms one hydrogen bond to the chloride anion and two hydrogen bonds to water mol­ecules. An intermolecular bond between the carbonyl O atom and the ammonium group [2.8459 (15) Å] is also found.

## Related literature

For the crystal structures of l-alanine and dl-alanine, see: Simpson & Marsh, (1966 ▶); Dunitz & Ryan, (1966 ▶); Lehmann *et al.* (1972 ▶); Destro *et al.* (1988 ▶); Donohue, (1950 ▶); Subha Nandhini *et al.* (2001 ▶). For the crystal structures of d-alanine hydro­chloride and dl-alanine hydro­chloride, see: di Blasio *et al.* (1977 ▶); Trotter, (1962 ▶). For the preparation of the title compound with respect to ^17^O-labelling, see: Steinschneider *et al.* (1981 ▶).
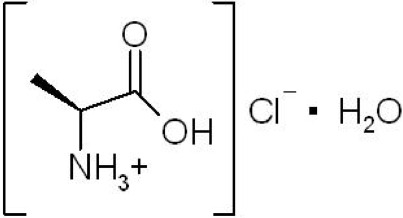


## Experimental

### 

#### Crystal data

C_3_H_8_NO_2_^+^·Cl^−^·H_2_O
*M* *_r_* = 143.57
Orthorhombic, 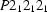

*a* = 6.1925 (13) Å
*b* = 9.929 (2) Å
*c* = 11.759 (3) Å
*V* = 723.0 (3) Å^3^
*Z* = 4
Mo *K*α radiation
μ = 0.46 mm^−1^
*T* = 150 (2) K
0.45 × 0.40 × 0.35 mm

#### Data collection

Bruker SMART APEX CCD area-detector diffractometer
Absorption correction: multi-scan (*SADABS*; Bruker 2001 ▶) *T*_min_ = 0.819, *T*_max_ = 0.855
5762 measured reflections
1467 independent reflections
1452 reflections with *I* > 2σ(*I*)
*R*_int_ = 0.022

#### Refinement

*R*[*F* ^2^ > 2σ(*F* ^2^)] = 0.019
*wR*(*F* ^2^) = 0.053
*S* = 1.11
1467 reflections
84 parameters
H atoms treated by a mixture of independent and constrained refinement
Δρ_max_ = 0.19 e Å^−3^
Δρ_min_ = −0.20 e Å^−3^
Absolute structure: Flack (1983 ▶), 533 Friedel pairs
Flack parameter: 0.02 (6)

### 

Data collection: *SMART for WNT/2000* (Bruker, 2001 ▶); cell refinement: *SAINT-Plus* (Bruker, 2001 ▶); data reduction: *SAINT-Plus*; program(s) used to solve structure: *SHELXS97* (Sheldrick, 2008 ▶); program(s) used to refine structure: *SHELXL97* (Sheldrick, 2008 ▶); molecular graphics: *ORTEP-3 for Windows* (Farrugia, 1997 ▶); software used to prepare material for publication: *SHELXL97*.

## Supplementary Material

Crystal structure: contains datablocks I, global. DOI: 10.1107/S1600536808008751/hg2382sup1.cif

Structure factors: contains datablocks I. DOI: 10.1107/S1600536808008751/hg2382Isup2.hkl

Additional supplementary materials:  crystallographic information; 3D view; checkCIF report

## Figures and Tables

**Table 1:** Hydrogen-bond geometry (Å, °)

*D*—H⋯*A*	*D*—H	H⋯*A*	*D*⋯*A*	*D*—H⋯*A*
N—H*A*⋯O3	0.89	1.96	2.8479 (14)	174
N—H*B*⋯Cl^i^	0.89	2.31	3.1957 (11)	171
N—H*C*⋯O3^ii^	0.89	1.95	2.8380 (15)	180
O2—H2⋯Cl	0.82	2.23	3.0446 (11)	175
O3—H4⋯Cl^iii^	0.82 (2)	2.35 (2)	3.1432 (12)	161.0 (17)
O3—H5⋯Cl^iv^	0.78 (2)	2.38 (2)	3.1283 (11)	163.3 (16)

Symmetry codes: (i) 
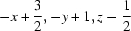
; (ii) 
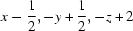
; (iii) 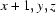; (iv) 
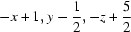
.

**Fig. 1.**
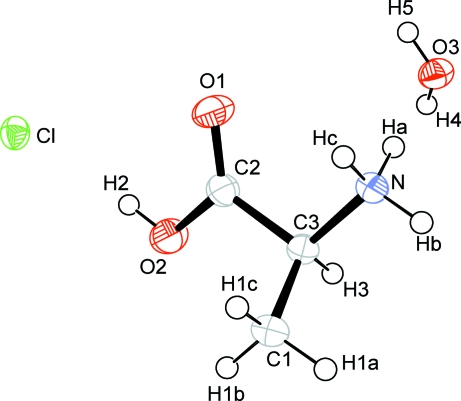
A view of the molecular structure of (I), showing the atom labeling scheme. Displacement ellipsoids are drawn at the 50% probability level.

**Fig. 2.**
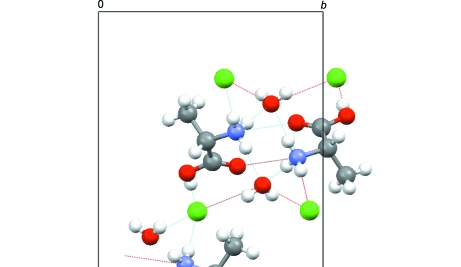
A packing diagram of (I) viewed from a axis. Broken lines indicate the hydrogen bonds.

## supplementary crystallographic information

### Comment

L-Alanine is one of the 20 proteinogenic amino acids and has been currently recognized as one of the most abundant amino acids in natural proteins. In general, amino acids very often have polymorphs. The crystal structures of L-alanine (Simpson & Marsh, 1966; Dunitz & Ryan, 1966; Lehmann *et al.*, 1972; Destro *et al.*, 1988), DL-alanine (Donohue, 1950; Subha Nandhini *et al.*, 2001; Trotter, 1962), and D-alanine hydrochloride (di Blasio *et al.*, 1977) have been reported. In the present study, a single-crystal structure determination of L-alanine hydrochloride monohydrate, (I), is reported.

The distances and angles of the present L-alanine molecule are consistent in the typical values reported in the literature of L-alanine molecules (See Table 1 and Figure 1). The atoms N, C2, C3, O1, and O2 are found to be nearly coplanar. The torsion angles of O2—C2—C3—N and O1—C2—C3—N are 174.27 (9) and -6.07 (18), respectively. The angles of O1—C2—O2 and O1—C2—C3, for example, are 125.35 (11) and 123.71 (11)°, respectively. These observations are in reasonable agreement with those of D-alanine hydrochloride reported previously (di Blasio *et al.*, 1977). The torsion angle of O2—C2—C3—C1 was observed to be -64.37 (14)°, which is slightly different from that of L-alanine, the corresponding angle was -76.0° (Lehmann *et al.*, 1972).

The single-crystal diffraction analysis exhibits that the titled compound contains several intermolecular hydrogen bonds. O2 is involved to a single hydrogen bond to a chloride ion with the hydrogen bond distance of 3.0446 (11) Å, while O1 is not involved to hydrogen bonds. Instead, a short contact is observed between O1 and N with the intermolecular bond length of 2.8450 (15) Å. A water molecule donates four hydrogen bonds to two chlorides and two ammonium groups, while a chloride ion accepts four hydrogen bonds from two water molecules, and ammonium and hydroxyl groups (See Table 2 and Figure 2).

It is interesting to compare the present structure with that of D-alanine hydrochloride (di Blasio *et al.*, 1977). In the anhydrous D-alanine hydrochloride crystal, O1 (carbonyl oxygen) exhibits a single hydrogen bond to an ammonium group, and O2 (hydroxyl oxygen) also forms a single hydrogen bond to a chloride ion. The chloride anion, on the other hand, forms three hydrogen bonds, two of which with ammonium groups and one of which with a hydroxyl group. These intermolecular environments are different from the present observations.

### Experimental

In the title compound, oxygen-17 isotope enrichments were carried out to the carboxyl group with the aim to perform solid-state ^17^O NMR experiments. L-alanine hydrochloride was obtained by acid-catalyzed exchange (Steinschneider *et al.*, 1981) with L-alanine and H_2_^17^O (20% ^17^O atom, purchased from Taiyo Nippon Sanso Corp., Tokyo, Japan). Colorless crystals of the title compound were obtained from the powder sample after it was left standing in a refrigerator for a few years.

### Refinement

All the H atoms except for H4 and H5 were treated as riding atoms with the following distances: for the methyl C—H distance, C—H = 0.96 Å and *U*_iso_(H) = 1.5*U*_eq_(C); for the methyne C—H distance, C—H = 0.98Å and *U*_iso_(H) = 1.2*U*_eq_(C); for the hydroxyl O—H distance, O—H = 0.82Å and *U*_iso_(H) = 1.2*U*_eq_(O); for the ammonium N—H distance, N—H = 0.89Å and *U*_iso_(H) = 1.5*U*_eq_(N). The H4 and H5 atoms were found in difference density Fourier maps, and their positions and isotropic displacement parameters were freely refined.

It might be possible that some degree of racemization occurred since the titled compound had been placed in the refrigerator for a few years. The present diffraction measurements, however, exhibited negligible recemization (the Flack parameter = 0.02 (6)).

It should be noted that "Alert Level B (detecting a pseudo center of symmetry)" was generated by checkCIF/*PLATON* REPORT during the course of the publication check. The error may come from the fact that the chloride anions are close to a center of symmetry. The present experimental data certainly suggest non-centrosymmetric.

### Crystal data

**Table:**

C_3_H_8_NO_2_^+^·Cl^–^·H_2_O	*F*_000_ = 304
*M**_r_* = 143.57	*D*_x_ = 1.319 Mg m^−^^3^
Orthorhombic, *P*2_1_2_1_2_1_	Mo *K*α radiation λ = 0.71073 Å
Hall symbol: P 2ac 2ab	Cell parameters from 902 reflections
*a* = 6.1925 (13) Å	θ = 7.4–53.9º
*b* = 9.929 (2) Å	µ = 0.46 mm^−^^1^
*c* = 11.759 (3) Å	*T* = 150 (2) K
*V* = 723.0 (3) Å^3^	Plate, colourless
*Z* = 4	0.45 × 0.40 × 0.35 mm

### Data collection

**Table:**

Bruker SMART APEX CCD area-detector diffractometer	1467 independent reflections
Radiation source: fine-focus sealed tube	1452 reflections with *I* > 2σ(*I*)
Monochromator: graphite	*R*_int_ = 0.022
*T* = 293(2) K	θ_max_ = 27.0º
ω scans	θ_min_ = 2.7º
Absorption correction: multi-scan(SADABS; Bruker 2001)	*h* = −7→7
*T*_min_ = 0.819, *T*_max_ = 0.855	*k* = −11→12
5762 measured reflections	*l* = −14→14

### Refinement

**Table:**

Refinement on *F*^2^	Hydrogen site location: inferred from neighbouring sites
Least-squares matrix: full	H atoms treated by a mixture of independent and constrained refinement
*R*[*F*^2^ > 2σ(*F*^2^)] = 0.019	*w* = 1/[σ^2^(*F*_o_^2^) + (0.0308*P*)^2^ + 0.0901*P*] where *P* = (*F*_o_^2^ + 2*F*_c_^2^)/3
*wR*(*F*^2^) = 0.053	(Δ/σ)_max_ = 0.001
*S* = 1.11	Δρ_max_ = 0.19 e Å^−^^3^
1467 reflections	Δρ_min_ = −0.20 e Å^−^^3^
84 parameters	Extinction correction: none
Primary atom site location: structure-invariant direct methods	Absolute structure: Flack (1983), 533 Friedel pairs
Secondary atom site location: difference Fourier map	Flack parameter: 0.02 (6)

### Special details

**Table:**

Geometry. All e.s.d.'s (except the e.s.d. in the dihedral angle between two l.s. planes) are estimated using the full covariance matrix. The cell e.s.d.'s are taken into account individually in the estimation of e.s.d.'s in distances, angles and torsion angles; correlations between e.s.d.'s in cell parameters are only used when they are defined by crystal symmetry. An approximate (isotropic) treatment of cell e.s.d.'s is used for estimating e.s.d.'s involving l.s. planes.
Refinement. Refinement of *F*^2^ against ALL reflections. The weighted *R*-factor *wR* and goodness of fit *S* are based on *F*^2^, conventional *R*-factors *R* are based on *F*, with *F* set to zero for negative *F*^2^. The threshold expression of *F*^2^ > σ(*F*^2^) is used only for calculating *R*-factors(gt) *etc*. and is not relevant to the choice of reflections for refinement. *R*-factors based on *F*^2^ are statistically about twice as large as those based on *F*, and *R*- factors based on ALL data will be even larger.

### Fractional atomic coordinates and isotropic or equivalent isotropic displacement parameters (Å^2^)

**Table:**

	*x*	*y*	*z*	*U*_iso_*/*U*_eq_	
Cl	0.19971 (4)	0.55991 (3)	1.25180 (2)	0.02475 (10)	
C1	0.8057 (2)	0.60202 (13)	0.88317 (11)	0.0295 (3)	
H1A	0.9301	0.6167	0.8360	0.044*	
H1B	0.7483	0.6872	0.9073	0.044*	
H1C	0.6979	0.5540	0.8407	0.044*	
C2	0.6780 (2)	0.49201 (12)	1.06398 (10)	0.0249 (2)	
N	0.96092 (17)	0.38728 (10)	0.95162 (8)	0.0230 (2)	
HA	1.0116	0.3443	1.0124	0.034*	
HB	1.0676	0.4003	0.9021	0.034*	
HC	0.8577	0.3381	0.9193	0.034*	
O3	1.13229 (15)	0.26883 (10)	1.15289 (8)	0.0261 (2)	
O1	0.60730 (17)	0.38141 (9)	1.08180 (8)	0.0342 (2)	
O2	0.60094 (18)	0.60494 (10)	1.10708 (9)	0.0374 (2)	
H2	0.4967	0.5880	1.1476	0.056*	
C3	0.87031 (18)	0.51981 (12)	0.98686 (10)	0.0228 (2)	
H3	0.9801	0.5694	1.0299	0.027*	
H4	1.159 (3)	0.335 (2)	1.1930 (16)	0.047 (5)*	
H5	1.050 (3)	0.2271 (18)	1.1882 (14)	0.036 (5)*	

### Atomic displacement parameters (Å^2^)

**Table:**

	*U*^11^	*U*^22^	*U*^33^	*U*^12^	*U*^13^	*U*^23^
Cl	0.02114 (14)	0.02522 (15)	0.02790 (15)	0.00128 (9)	0.00012 (12)	−0.00516 (12)
C1	0.0308 (6)	0.0239 (6)	0.0337 (6)	0.0021 (5)	0.0026 (5)	0.0073 (5)
C2	0.0260 (5)	0.0230 (5)	0.0256 (5)	0.0013 (5)	0.0015 (5)	−0.0018 (5)
N	0.0232 (5)	0.0200 (5)	0.0257 (5)	0.0019 (4)	0.0028 (4)	0.0023 (4)
O3	0.0281 (5)	0.0235 (4)	0.0268 (5)	−0.0044 (4)	0.0023 (4)	−0.0008 (4)
O1	0.0398 (5)	0.0238 (4)	0.0389 (5)	−0.0059 (4)	0.0160 (4)	−0.0029 (4)
O2	0.0384 (5)	0.0244 (4)	0.0494 (6)	0.0011 (4)	0.0183 (5)	−0.0046 (4)
C3	0.0227 (5)	0.0183 (5)	0.0274 (6)	−0.0003 (4)	0.0011 (5)	−0.0002 (4)

### Geometric parameters (Å, °)

**Table:**

C1—C3	1.5209 (17)	N—HA	0.8900
C1—H1A	0.9600	N—HB	0.8900
C1—H1B	0.9600	N—HC	0.8900
C1—H1C	0.9600	O3—H4	0.82 (2)
C2—O1	1.2006 (16)	O3—H5	0.78 (2)
C2—O2	1.3196 (15)	O2—H2	0.8200
C2—C3	1.5223 (16)	C3—H3	0.9800
N—C3	1.4893 (15)		
			
C3—C1—H1A	109.5	C3—N—HC	109.5
C3—C1—H1B	109.5	HA—N—HC	109.5
H1A—C1—H1B	109.5	HB—N—HC	109.5
C3—C1—H1C	109.5	H4—O3—H5	104.4 (17)
H1A—C1—H1C	109.5	C2—O2—H2	109.5
H1B—C1—H1C	109.5	N—C3—C1	110.50 (10)
O1—C2—O2	125.34 (11)	N—C3—C2	107.48 (9)
O1—C2—C3	123.71 (11)	C1—C3—C2	111.65 (11)
O2—C2—C3	110.95 (10)	N—C3—H3	109.1
C3—N—HA	109.5	C1—C3—H3	109.1
C3—N—HB	109.5	C2—C3—H3	109.1
HA—N—HB	109.5		
			
O1—C2—C3—N	−6.06 (18)	O1—C2—C3—C1	115.28 (14)
O2—C2—C3—N	174.26 (9)	O2—C2—C3—C1	−64.39 (14)

### Hydrogen-bond geometry (Å, °)

**Table:**

*D*—H···*A*	*D*—H	H···*A*	*D*···*A*	*D*—H···*A*
N—HA···O3	0.89	1.96	2.8479 (14)	174
N—HB···Cl^i^	0.89	2.31	3.1957 (11)	171
N—HC···O3^ii^	0.89	1.95	2.8380 (15)	180
O2—H2···Cl	0.82	2.23	3.0446 (11)	175
O3—H4···Cl^iii^	0.82 (2)	2.35 (2)	3.1432 (12)	161.0 (17)
O3—H5···Cl^iv^	0.78 (2)	2.38 (2)	3.1283 (11)	163.3 (16)

Symmetry codes: (i) −*x*+3/2, −*y*+1, *z*−1/2; (ii) *x*−1/2, −*y*+1/2, −*z*+2; (iii) *x*+1, *y*, *z*; (iv) −*x*+1, *y*−1/2, −*z*+5/2.
